# RSMA-enabled satellite-terrestrial communication networks: Performance analysis

**DOI:** 10.1371/journal.pone.0357013

**Published:** 2026-08-26

**Authors:** Hong-Nhu Nguyen, Quang Sang Nguyen, Tan N. Nguyen

**Affiliations:** 1 Faculty of Technology and Engineering, Saigon University (SGU), Ho Chi Minh City, Vietnam; 2 Posts and Telecommunications Institute of Technology, Ho Chi Minh City, Vietnam; 3 Advanced Intelligent Technology Research Group, Faculty of Electrical and Electronics Engineering, Ton Duc Thang University, Ho Chi Minh City, Vietnam; Lincoln University College, MALAYSIA

## Abstract

This paper investigates the performance of a satellite-terrestrial communication system employing rate-splitting multiple access (RSMA) to accommodate multiple users. The study focuses on assessing the system’s ergodic sum rate and symbol error rate (SER) under shadowed-Rician fading conditions. By deriving closed-form expressions, we analyze the influence of various power allocation strategies on overall system performance. The results, substantiated by numerical simulations, demonstrate that RSMA yields significant improvements over conventional non-orthogonal multiple access (NOMA) with respect to spectral efficiency and interference management. These findings offer valuable insights for the development of more reliable and efficient satellite-terrestrial networks.

## 1 Introduction

Hybrid Satellite–Terrestrial Networks (HSTNs) are increasingly vital in contemporary wireless systems, providing extensive coverage and dependable connectivity in remote and under-served regions. These networks represent a pivotal technology in the progression of next-generation communication systems, including 6G. HSTNs provide a comprehensive framework for addressing both technological advancements and practical challenges inherent, thereby establishing themselves as a foundational component for the seamless integration of terrestrial and non-terrestrial networks in the 6G era and beyond [1]. With the swift rise in data traffic and the demand for enhanced spectral efficiency, traditional multiple-access methods encounter considerable obstacles in accommodating the growing user base and effectively managing interference [2,3]. To improve spectral efficiency, orthogonal multiple access (OMA) is commonly utilized in satellite communication systems, where distinct frequency, time, or code resources are allocated to different users to prevent interference [4]. Nonetheless, OMA is limited in its spectral utilization, especially when faced with a high number of users [5]. To address this drawback, non-orthogonal multiple access (NOMA) has been developed, enabling multiple users to utilize the same resources through power-domain multiplexing [6,7]. Although NOMA enhances spectral efficiency by facilitating successive interference cancellation (SIC) at the receiver, it also presents challenges like error propagation, issues of fairness, and heightened decoding complexity [8,9]. Recently, Rate-Splitting Multiple Access (RSMA) has emerged as a favorable alternative, encompassing both OMA and NOMA [10]. Unlike NOMA, which depends on SIC for separating signals, RSMA divides transmitted messages into common and private segments, enabling more adaptable interference management [11,12]. The common message is available for all users to decode, while private messages are decoded separately, striking a balance between interference mitigation and spectral efficiency [13]. Research indicates that RSMA surpasses both OMA and NOMA regarding sum rate, user fairness, and resilience to imperfect channel state information (CSI) [14–16]. However, although RSMA has been thoroughly studied in terrestrial networks, its application in satellite-terrestrial communication is still largely uncharted, particularly under realistic fading conditions like shadowed-Rician fading [17].

### 1.1 Literature review

Since its introduction, RSMA has gained significant research attention, with numerous studies focusing on resource allocation optimization and precoder design to enhance user rate fairness, maximize sum rate, and improve bit error rate (BER) and symbol error rate (SER) performance. For instance, the work in [18] explored a joint optimization framework for rate-splitting precoding, subcarrier assignment, and power allocation to ensure fair rate distribution among users in multi-cast transmissions. To further analyze BER performance under finite constellation and specific coding rates, a transceiver architecture was proposed, incorporating low-density parity-check (LDPC) encoding, modulation, and RS-based signal splitting at the transmitter, along with maximum a posteriori (MAP) detection, LDPC decoding, and SIC at the receiver. Additionally, in [19], researchers examined half-duplex cooperative communication (HCC) for RSMA users, demonstrating that even with partial CSI, jointly optimizing the precoder, time allocation, and relay node selection could still achieve a higher weighted sum rate (WSR) compared to non-cooperative communication (NCC). Existing studies can therefore be broadly categorized into two main methodological directions. Regarding the first methodology, which avoids the use of a precoder, the majority of research concentrates on utilizing the inherent advantages of rate-splitting techniques to enhance capacity. For example, the study in [20] introduced fixed and cognitive rate splitting strategies employing fixed and cyclic single-carrier transmissions to improve the fairness of pairing nearby users with distant users while also reducing their overall outage probability in delay-constrained transmission scenarios. In [21], a novel reconfigurable intelligent surface (RIS) rate-splitting framework was created utilizing an offline control mechanism to minimize outage occurrences for user communication. In [22], the application of rate-splitting for Low Earth Orbit (LEO) satellite communication (SatCom) was investigated with the objective of maximizing the sum rate of LEO SatCom systems. This was achieved through the simultaneous optimization of the power budget across multiple beams, RSMA power allocation for users within each beam, and subcarrier user assignment. Additionally, measures were implemented to ensure that interference temperature constraints to GEO SatCom were strictly maintained. Specifically, the research presented in [23] examined the outage probability and ergodic capacity approximations for MIMO systems while accounting for ULA and imperfect CSI, alongside a simulated assessment with various types of phase-shift keying (PSK) and quadrature amplitude modulation. In the context of downlink multiple-input single-output (MISO) networks featuring cell-edge users supported by reconfigurable intelligent surfaces and LRW precoders, the outage probability was extensively analyzed with a two-layer hierarchical resource sharing approach employing an on-off control method in [24] and a singular RS layer integrated with discrete phase shifters in [25].

### 1.2 Motivations and contributions

While RSMA has been thoroughly examined within multi-antenna and massive MIMO frameworks, its application to multi-user satellite-terrestrial communication systems remains insufficiently explored [26,27]. Additionally, existing research on RSMA often assumes the availability of perfect CSI, which is difficult to obtain in practical satellite networks due to significant mobility and beam alignment challenges [28]. To tackle this concern, this paper offers an in-depth analysis of RSMA-based satellite-terrestrial communication systems, focusing on ergodic sum rate and SER results under realistic fading conditions. The main contributions of this study are summarized as follows:

- Ergodic Sum Rate Analysis: We establish a closed-form integral expression for the ergodic sum rate in RSMA-driven satellite-terrestrial networks. A high-SNR approximation is included to provide a further understanding of system behavior at elevated transmit power levels.
- SER Evaluation: We evaluate the SER performance of both common and private messages during RSMA transmission. The influence of power allocation and modulation techniques (e.g., BPSK, QPSK) on SER is explored.
- Impact of Power Allocation and Fading Conditions: We investigate how the power coefficient for the common stream affects system performance, particularly under shadowed-Rician fading.
- Comparison with NOMA: We perform numerical simulations to juxtapose RSMA and traditional NOMA, emphasizing RSMA’s benefits in spectral efficiency and interference handling.

From an implementation standpoint, RSMA generally entails lower and more predictable receiver complexity than power-domain NOMA in satellite downlinks. Each user decodes the common stream once, cancels it via a single SIC step, and then decodes its private stream, so the SIC depth per terminal remains essentially constant. In contrast, NOMA typically requires successive decoding of multiple users’ signals (especially at strong users), which increases decoding latency and can amplify error propagation. While RSMA requires optimization of splitting and power allocation, such processing can be centralised (e.g., at the gateway), whereas NOMA additionally relies on user ordering/pairing decisions and related signalling overhead.

The outcomes of this research provide critical insights that are necessary for improving RSMA-based satellite-terrestrial networks. These findings directly contribute to the development of more efficient and robust next-generation satellite communication systems.

### 1.3 Organization & notations

The remainder of this paper is organized as follows: Section 2 introduces the system model, explaining the satellite-terrestrial channel characteristics and the RSMA transmission framework. Section 3 focuses on the derivation of the ergodic sum rate and its implications for system performance. Section 4 analyzes the SER, considering different power allocation strategies. Section 5 presents numerical results, comparing RSMA with conventional multiple access schemes and discussing key observations. Finally, Section 6 concludes the paper and suggests directions for future research.

For clarity, the key notations used throughout this paper are summarized in Table 1.

**Table 1:** Summary of Key Notations.

Notation	Description
*K*, *Q*	Number of satellite antennas and terrestrial users, respectively
$S,U_{q}$	The GEO satellite and the *q*-th user
${𝐡}_{U_{q}},{𝐠}_{U_{q}}$	Channel gain and Shadowed-Rician channel vector for $U_{q}$
${𝐰}_{U_{q}}$	Transmit beamforming vector for $U_{q}$
$P_{S},\varrho_{S}$	Total transmit power and transmit SNR at the satellite *S*
$x_{c},x_{q}$	Common message and private message for $U_{q}$
$a_{c},a_{q}$	Power allocation coefficients for $x_{c}$ and $x_{q}$
${\bar{\gamma }}_{c,q},{\bar{\gamma }}_{p,q}$	SINR for decoding the common and private messages at $U_{q}$
$\delta_{q}$	Scaling factor including path loss and antenna gain $\delta_{q}=\varrho_{S}L_{SU_{q}}\vartheta_{S}\vartheta (\theta_{U_{q}})$
${𝒜}_{q}$	Effective channel gain component, ${𝒜}_{q}=\delta_{q}\|{𝐠}_{U_{q}}{\|}_{F}^{2}$
$\psi_{q}$	Auxiliary fading parameter defined as $\psi_{q}=\beta_{q}-\delta_{q}$
$\Delta_{q}$	Auxiliary parameter defined as $\sum_{l=1}^{K}j_{l}+K$
$R_{c,q},R_{p,q}$	Ergodic rate of the common and private streams at $U_{q}$
$S_{c,q},S_{p,q}$	Symbol error rate (SER) of the common and private messages at $U_{q}$
$I_{i}(\cdot ){,}_{1}F_{1}(\cdot )$	Modified Bessel function of the first kind and confluent hypergeometric function
$ℬ(\cdot ),\gamma (\cdot ),G_{p,q}^{m,n}[\cdot ]$	Beta function, lower incomplete Gamma function, and Meijer’s G-function
*Q*(*x*), $(\cdot {)}_{t}$	Gaussian Q-function and Pochhammer symbol

## 2 System model

### 2.1 System description

**Fig 1:**
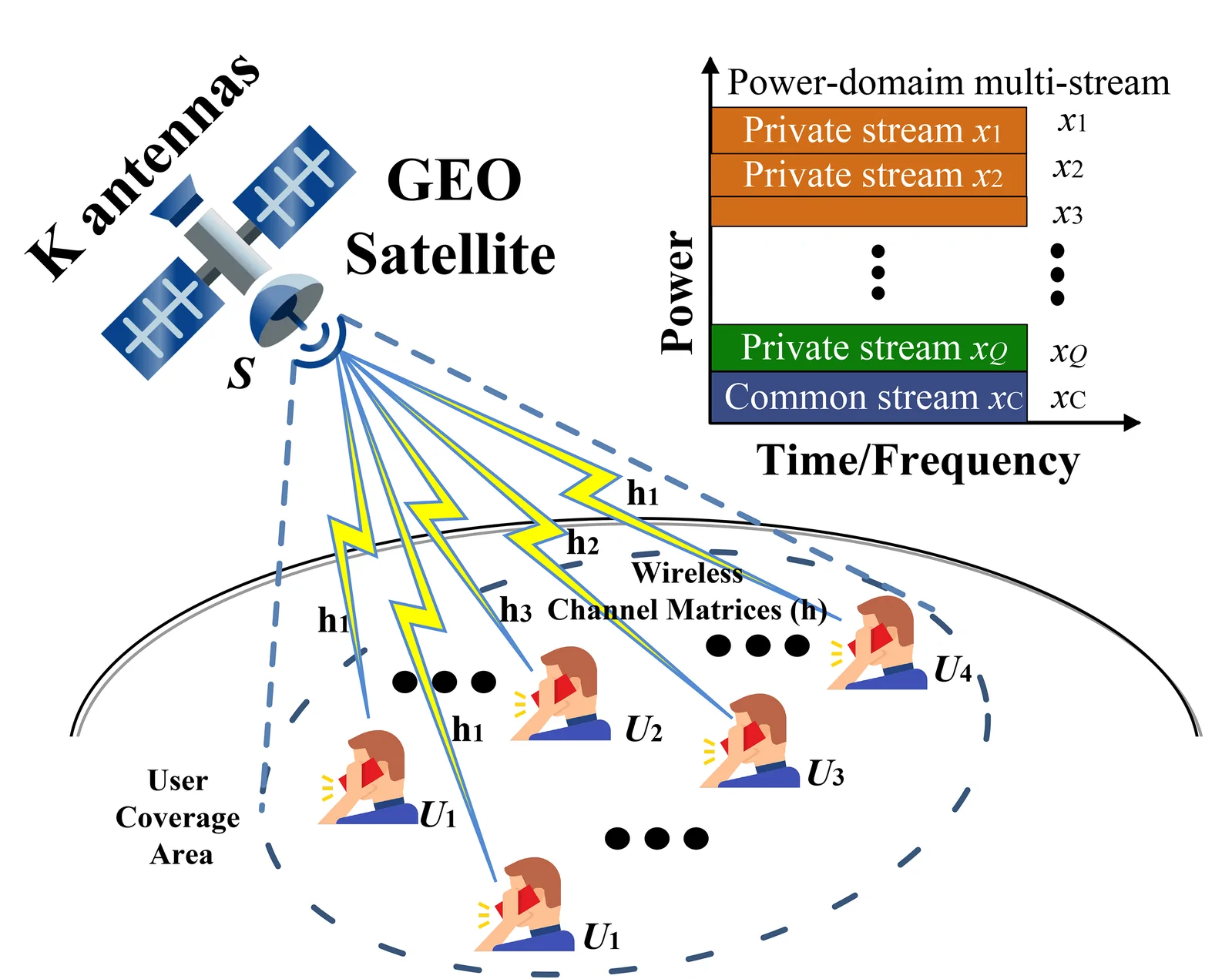
An illustration of a downlink RSMA-enabled satellite-terrestrial communication system.

Fig 1 illustrates the considered downlink RSMA-enabled satellite–terrestrial system. A GEO satellite *S* equipped with *K* antennas simultaneously serves *Q* single-antenna terrestrial users ${\left\{U_{q}\right\}}_{q=1}^{Q}$. The satellite superposes a common stream $x_{c}$ and private stream $\left\{x_{q}\right\}$ with power fractions $a_{c}$ and $\left\{a_{q}\right\}$, respectively, under the total power constraint $a_{c}+\sum_{q=1}^{Q}a_{q}=1$. The effective channel to user $U_{q}$ is $h_{U_{q}}=g_{U_{q}}^{†}w_{U_{q}}\sqrt{L_{SU_{q}}\vartheta_{S}\vartheta \left(\theta_{U_{q}}\right)}$, where $g_{U_{q}}$ follows a shadowed-Rician model, $w_{U_{q}}$ is selected according to MRT, and $L_{SU_{q}}$ and $\vartheta (\theta_{U_{q}})$ capture the free-space loss and satellite beam gain, respectively. Each user decodes $x_{c}$ first while treating all private streams as interference, performs SIC to remove $x_{c}$ and then decodes its own private stream in equation (4), leading to the SINRs in equations (5) and (6). Unless otherwise stated, we adopt ideal (benchmark) CSI at the transmitter for MRT beamforming and analysis tractability, as discussed in this section.

Modern satellite systems often use multi-beam technology to enhance coverage and capacity. For geosynchronous Earth orbit (GEO) satellites, these beams are usually generated by array-fed reflectors, which offer better efficiency compared to direct radiating arrays. The beam pattern remains fixed, reducing the need for complex onboard processing.

The signal received by each user depends on the satellite’s beamforming strategy, propagation conditions, and power allocation. The channel gain between the satellite and the *q*th user is given by


$$\begin{array}{c} h_{U_{q}}=g_{U_{q}}^{†}w_{U_{q}}\sqrt{L_{SU_{q}}\vartheta_{S}\vartheta \left(\theta_{U_{q}}\right)}, \end{array}$$ (1)

where $\vartheta_{S}$ represents the satellite antenna gain, and $g_{U_{q}}$ denotes the K×1 Shadowed-Rician channel vector linking *K* antennas at *S*. The notation ${\left(.\right)}^{†}$ indicates the conjugate transpose, and $w_{U_{q}}$ is the K×1 vector of transmit weights. The transmit beamforming vector $w_{U_{q}}\in {\mathbb{C}}^{K\times 1}$ is chosen based on the maximum ratio transmission (MRT) principle, such that $w_{U_{q}}=\frac{\left\|g_{U_{q}}\right\|}{{\left\|g_{U_{q}}\right\|}_{F}}$, where ${\left\|.\right\|}_{F}$ indicates the Frobenius norm. Additionally, $L_{SU_{q}}=\frac{1}{K_{B}TW}{\left(\frac{c}{4\pi f_{c}d_{SU_{q}}}\right)}^{2}$ represents the instantaneous free space loss [29], where $\frac{1}{K_{B}TW}$ serves as a scaling factor, with $K_{B}=1.38\times 10^{-23}\;J\;\cdot K$ designated as the Boltzmann constant, *T* as the noise temperature of the receiver, *W* as the bandwidth of the carrier, *c* as the speed of light, $f_{c}$ as the frequency of the carrier, and $d_{SU_{q}}$ as the distance separating *S* and $U_{q}$. Furthermore, the beam gain $\vartheta \left(\theta_{U_{q}}\right)$ of the satellite can be expressed as


$$\begin{array}{c} \vartheta \left(\theta_{U_{q}}\right)=\vartheta_{U_{q}}\left(\frac{I_{1}\left({\bar{\rho }}_{U_{q}}\right)}{2{\bar{\rho }}_{U_{q}}}+36\frac{I_{3}\left({\bar{\rho }}_{U_{q}}\right)}{{\bar{\rho }}_{U_{q}}^{3}}\right), \end{array}$$ (2)

where $I_{i}\left(.\right)$ is the first-kind Bessel function with order *i*, $\theta_{U_{q}}$ is the antenna gain at $U_{q}$, $\theta_{U_{q}}$ is the angular separation and ${\bar{\rho }}_{U_{q}}=2.07123\frac{\sin\theta_{U_{q}}}{\sin\theta_{U_{q}3dB}}$ in which $\sin\theta_{U_{i}3dB}$ represents the 3 dB beam-width. Furthermore, it is assumed that the ideal CSI $g_{U_{q}}$ is accessible to the satellite user. This can be achieved by using training symbols for the satellite connection, together with an existing feedback link or spectrum manager (which acts as an intermediary between the two systems) for the terrestrial interference link (Perfect CSI is assumed to obtain a tractable analytical benchmark and to highlight the intrinsic RSMA gains under shadowed-Rician satellite fading. In practice, CSI acquisition in satellite–terrestrial links is affected by estimation errors, feedback quantization, and latency. For GEO multi-beam downlinks, the relatively slow variation of the effective channel and the availability of pilot-assisted estimation with a return link (or a spectrum manager) can make the CSI quality sufficiently high to approximate the perfect-CSI benchmark. For more dynamic scenarios (e.g., LEO or rapid beam tracking), imperfect CSI models are required; robust RSMA designs under CSI uncertainty constitute an important extension and will be considered in future work.) [30].

### 2.2 The signal processing at transceivers

In this system, the satellite uses RSMA to transmit signals to multiple users at the same time. The transmitted signal consists of two parts: a common message that is decoded by all users and private messages intended for individual users. To balance performance, the satellite allocates power to these messages using a power coefficient $a_{c}$ for the common message, while the remaining power is distributed among the private messages.

The transmitted signal can be expressed as follows:


$$\begin{array}{c} x=\sqrt{P_{S}}\left(\sqrt{a_{c}}x_{c}+\sum_{q=1}^{Q}\sqrt{a_{q}}x_{q}\right), \end{array}$$ (3)

Here $P_{S}$ is the total transmit power at S, which $x_{c}$ is the common message, allocated with power $a_{c}P_{S}$ and $x_{q}$ is the private message for user *q*, allocated with power $a_{q}P_{S}$. It is important to note that $a_{c}+\sum_{q=1}^{Q}a_{q}=1$. The signal obtained by the *q*th user can be articulated as


$$\begin{array}{cc} y_{U_{q}}= & h_{U_{q}}x+n_{U_{q}}\\ = & \underset{\begin{array}{c} Common\;Message \end{array}}{\underbrace{g_{U_{q}}^{†}w_{U_{q}}\sqrt{L_{SU_{q}}\vartheta_{S}\vartheta \left(\theta_{U_{q}}\right)a_{c}P_{S}}x_{c}}}+\underset{\begin{array}{c} Desired\;Private\;Message \end{array}}{\underbrace{g_{U_{q}}^{†}w_{U_{q}}\sqrt{L_{SU_{q}}\vartheta_{S}\vartheta \left(\theta_{U_{q}}\right)a_{q}P_{S}}x_{q}}}\\ & +\underset{\begin{array}{c} Interference \end{array}}{\underbrace{\sum_{j=1,j\neq q}^{Q}g_{U_{q}}^{†}w_{U_{q}}\sqrt{L_{SU_{q}}\vartheta_{S}\vartheta \left(\theta_{U_{q}}\right)a_{j}P_{S}}x_{j}}}+\underset{\begin{array}{c} AWGN \end{array}}{\underbrace{n_{U_{q}}}}, \end{array}$$ (4)

Here $n_{U_{q}}$ represents the additive white Gaussian noise (AWGN) characterized by a mean of zero and a variance denoted by $\sigma_{q}^{2}$. To decode the received signal, each user follows a two-step process.

### 2.3 Decoding the common message

Each user first decodes the common message $x_{c}$ while treating all private messages as interference. The signal-to-interference-plus-noise ratio (SINR) for decoding the common message at the user *q* is:


$$\begin{array}{cc} {\bar{\gamma }}_{c,q}= & \frac{a_{c}\delta_{q}P_{S}{\left\|g_{U_{q}}\right\|}_{F}^{2}}{\delta_{q}P_{S}\left(1-a_{c}\right){\left\|g_{U_{q}}\right\|}_{F}^{2}+\sigma_{q}^{2}}=\frac{a_{c}A_{q}}{\left(1-a_{c}\right)A_{q}+1}, \end{array}$$ (5)

where $\varrho_{S}=P_{S}\cdot \sigma_{q}^{2}$ is the transmit signal-to-noise ratio (SNR), while $A_{q}=\delta_{q}{\left\|g_{U_{q}}\right\|}_{F}^{2}$ and $\delta_{q}=\varrho_{S}L_{SU_{q}}\vartheta_{S}\vartheta \left(\theta_{U_{q}}\right)$.

### 2.4 Decoding the private message

Once the common message is successfully decoded and removed from the received signal, each user proceeds to decode its own private message. The remaining interference comes from private messages intended for other users. The SINR for decoding the private message is given by


$$\begin{array}{c} {\bar{\gamma }}_{p,q}=\frac{a_{q}A_{q}}{A_{q}\sum_{j=1,q\neq j}^{Q}a_{j}+1}. \end{array}$$ (6)

The efficiency of this process depends on the power allocation and channel conditions. By carefully choosing $a_{c}$ and $a_{q}$, RSMA ensures a balance between spectral efficiency and interference management, improving overall system performance.

Having established the signal model and the RSMA decoding procedure, we briefly discuss key practical implementation considerations before introducing the channel model used for performance analysis. Practical deployment of RSMA in satellite-terrestrial networks entails several implementation challenges. First, multi-antenna transmission relies on sufficiently accurate and timely CSI to support beamforming; in operational systems, CSI is typically acquired via pilot-assisted estimation and delivered through a feedback/coordination mechanism, and it may be affected by latency, quantization, and beam pointing errors. In this work, we adopt ideal CSI as a benchmark to enable MRT beamforming and to keep the analysis tractable, while acknowledging that imperfect CSI modeling and robust design are important extensions. Second, RSMA requires adaptive configuration of the common/private power split $\left(a_{c},\left\{a_{q}\right\}\right)$ and, in general, the allocation of the common rate across users, which can impose additional control-plane signaling and scheduling overhead, particularly when users experience heterogeneous channel conditions. Third, although the receiver-side processing in RSMA has relatively bounded complexity—each user decodes the common stream, removes it via SIC, and then decodes its private stream—non-ideal SIC and practical receiver impairments can still lead to residual interference and performance loss. Finally, satellite system constraints (e.g., payload processing capability, feeder-link limitations, and beam management) may restrict the update rate and granularity of precoding and power control, making low-complexity or gateway-centric implementations especially relevant. Motivated by these practical considerations, the following subsection formalizes the satellite channel statistics used in our performance analysis.

### 2.5 Terrestrial channel model

To facilitate the computation of system performance metrics, we assume that the channel coefficients are independent and identically distributed (i.i.d.). Under this assumption, the probability density function (PDF) of the channel coefficient $g_{q}\left(k\right)$, for all q∈Q, representing the link from the satellite’s *k*th antenna to the $q^{th}$ user, can be expressed as follows:


$$\begin{array}{c} \begin{array}{cc} f_{{\left|g_{q}^{\left(k\right)}\right|}^{2}}\left(x\right)=\alpha_{q}{e^{-\beta_{q}x}}_{1}F_{1}\left(m_{q};1;\varpi_{q}x\right) & ,x\geq 0 \end{array}, \end{array}$$ (7)

Where: $\alpha_{q}={\left(2b_{q}m_{q}/\left(2b_{q}m_{q}+\Omega_{q}\right)\right)}^{m_{q}}/2b_{q}$, $\;\beta_{q}=1/2b_{q}$, $\varpi_{q}=\Omega_{q}/\left(2b_{q}\right)\left(2b_{q}m_{q}+\Omega_{q}\right)$ in which Ωq, 2*bq*, and *mq* represent the average power of the line-of-sight (LOS) component, the average power of the multipath components, and the fading severity parameter, respectively. ${\;}_{1}F_{1}\left(\cdot ;\cdot ;\cdot \right)$ denotes a confluent hypergeometric function of the first kind, as referenced in [31, Eq. (9.210.1)]. For the purposes of this paper, we assume integer values for the Shadowed-Rician fading severity parameter $m_{q}$. This assumption facilitates a more straightforward analysis of the channel’s statistical characteristics and their influence on system performance metrics. Accordingly, equation (7) can be reformulated as follows:


$$\begin{array}{c} \begin{array}{cc} f_{{\left|g_{q}^{\left(k\right)}\right|}^{2}}\left(x\right)=\alpha_{q}e^{-\left(\beta_{q}-\varpi_{q}\right)x}\sum_{t=0}^{m_{q}-1}\zeta_{q}\left(t\right)x^{t} & ,x\geq 0 \end{array}, \end{array}$$ (8)

where $\zeta_{q}(t)=(-1{)}^{t}(1-m_{q}{)}_{t}\varpi_{q}^{t}/(t!{)}^{2}$ and ${\left(.\right)}_{t}$ denotes the Pochhammer symbol. Drawing upon the findings presented in [32], the PDF of Aq under independent and identically distributed (i.i.d.) Shadowed-Rician fading can be expressed as follows:


$$\begin{array}{c} f_{A_{q}}\left(x\right)=\sum_{j_{1}=0}^{m_{q}-1}\cdots \sum_{j_{K}=0}^{m_{q}-1}\frac{\Lambda_{q}\left(K\right)}{\delta_{q}^{\Delta_{q}}}x^{\Delta_{q}-1}e^{-\left(\frac{\psi_{q}}{\delta_{q}}\right)x}, \end{array}$$ (9)

where


$$\begin{array}{c} \begin{array}{c} \Lambda_{q}\left(K\right)=\alpha_{q}^{K}\prod_{l=1}^{K}\zeta_{q}\left(j_{l}\right)\prod_{u=1}^{K-1}B\left(\sum_{p=1}^{u}j_{p}+u,j_{u+1}+1\right), \end{array} \end{array}$$ (10)

In this context,


$$\begin{array}{c} \Delta_{q}=\sum_{l=1}^{K}j_{l}+K,\;\;\;\psi_{q}=\beta_{q}-\delta_{q}, \end{array}$$ (11)

The Beta function B(.,.) is referenced from [31, Eq. (8.384.1)]. To obtain the CDF of γi, we directly utilize the result from [31, Eq. (3.351.1)]:


$$\begin{array}{c} F_{A_{q}}\left(x\right)=\sum_{j_{1}=0}^{m_{q}-1}\cdots \sum_{j_{K}=0}^{m_{q}-1}\frac{\Lambda_{q}\left(K\right)}{\psi_{q}^{\Delta_{q}}}\gamma \left(\Delta_{q},\frac{\psi_{q}x}{\delta_{q}}\right). \end{array}$$ (12)

where γ(.,.) is the lower incomplete Gamma function. Using [31, Eq. (8.352.6)], (12) can be simplified as


$$\begin{array}{c} F_{A_{q}}\left(x\right)=1-\sum_{j_{1}=0}^{m_{q}-1}\cdots \sum_{j_{K}=0}^{m_{q}-1}\sum_{p=0}^{\Delta_{q}-1}\frac{\Lambda_{q}\left(K\right)\Gamma \left(\Delta_{q}\right)}{p!\psi_{q}^{\Delta_{q}-p}\delta_{q}^{p}}e^{-\frac{\psi_{q}x}{\delta_{q}}}x^{p}. \end{array}$$ (13)

*Remark 1:* This formulation captures the statistical properties of the channel and facilitates a comprehensive understanding of the system’s performance metrics under the designated fading conditions.

## 3 Integral expression of ergodic sum rate

In terms of the instantaneous SINR, the ergodic rate of the common/private stream for the *q*th user can be given as [33,34]


$$\begin{array}{cc} R_{a,q}= & 𝔼\left[\log_{2}\left({\bar{\gamma }}_{a,q}+1\right)\right]=\frac{1}{\ln2}\int_{0}^{\infty }\frac{\left[1-F_{{\bar{\gamma }}_{a,q}}\left(x\right)\right]}{1+x}dx, \end{array}$$ (14)

where $R_{a,q}$ is normalized in the unit of bps/Hz. *a* = *c* denotes the common stream, whereas *a* = *p* denotes the private stream.

The common stream carries information intended to be decoded by all users at the receiver side. To guarantee that the shared stream can be interpreted by every user, the ergodic rate of the shared stream for each user must meet


$$\begin{array}{cc} & R_{c}=\min\left(R_{1,c},R_{2,c},...,R_{Q,c}\right), \end{array}$$ (15)

$$\begin{array}{cc} & \sum_{q=1}^{Q}C_{q}=R_{c}. \end{array}$$ (16)

In the satellite-terrestrial communication system that employs RSMA, the ergodic sum rate for the *q*th user is the total of the common rate and the private rate, which can therefore be expressed as


$$\begin{array}{cc} R_{sum}= & \sum_{q=1}^{Q}\left(C_{q}+R_{p,q}\right)=\min\left(R_{1,c},R_{2,c},...,R_{Q,c}\right)+\sum_{q=1}^{Q}R_{p,q}. \end{array}$$ (17)

*Proposition 1:* The ergodic sum rate may be accurately expressed as follows:


$$\begin{array}{cc} & R_{sum}≃\underset{q\in \left\{1,2,...,Q\right\}}{\min}\frac{1}{\ln2}\sum_{j_{1}=0}^{m_{q}-1}\cdots \sum_{j_{K}=0}^{m_{q}-1}\sum_{p=0}^{\Delta_{q}-1}\frac{\Lambda_{q}\left(K\right)\Gamma \left(\Delta_{q}\right)}{p!\psi_{q}^{\Delta_{q}-p}\delta_{q}^{p}}\\ & \times \left[B_{1}^{p}G_{1,2}^{2,1}\left(\frac{\psi_{q}B_{1}}{\delta_{q}}|\begin{array}{c} -p\\ -p,0 \end{array}\right)-B_{2}^{p}G_{1,2}^{2,1}\left(\frac{\psi_{q}B_{2}}{\delta_{q}}|\begin{array}{c} -p\\ -p,0 \end{array}\right)\right]\\ & +\frac{1}{\ln2}\sum_{q=1}^{Q}\frac{1}{\ln2}\sum_{j_{1}=0}^{m_{q}-1}\cdots \sum_{j_{K}=0}^{m_{q}-1}\sum_{p=0}^{\Delta_{q}-1}\frac{\Lambda_{q}\left(K\right)\Gamma \left(\Delta_{q}\right)}{p!\psi_{q}^{\Delta_{q}-p}\delta_{q}^{p}}\\ & \times \left[B_{3}^{p}G_{1,2}^{2,1}\left(\frac{\psi_{q}B_{3}}{\delta_{q}}|\begin{array}{c} -p\\ -p,0 \end{array}\right)-B_{4}^{p}G_{1,2}^{2,1}\left(\frac{\psi_{q}B_{4}}{\delta_{q}}|\begin{array}{c} -p\\ -p,0 \end{array}\right)\right], \end{array}$$ (18)

$B_{1}={\left[a_{c}+\left(1-a_{c}\right)\right]}^{-1}$, $B_{2}={\left(1-a_{c}\right)}^{-1}$, $B_{3}={\left[a_{q}+\left(1-a_{c}-a_{q}\right)\right]}^{-1}$ and $B_{4}=a_{q}^{-1}$.

*Proof:* First, substituting (5) into (14) we have the ergodic rate for decoding common messages is given by


$$\begin{array}{cc} R_{c,q}= & 𝔼\left[\log_{2}\left({\bar{\gamma }}_{c,q}+1\right)\right]=𝔼\left[\log_{2}\left(\underset{X}{\underbrace{\frac{a_{c}A_{q}}{\left(1-a_{c}\right)A_{q}+1}}}+1\right)\right]\\ = & \frac{1}{\ln2}\int_{0}^{\infty }\frac{\left[1-F_{X}\left(x\right)\right]}{1+x}dx=\frac{1}{\ln2}\int_{0}^{\frac{a_{c}}{1-a_{c}}}\frac{1}{1+x}\left[1-F_{A_{q}}\left(\frac{x}{a_{c}-\left(1-a_{c}\right)x}\right)\right]dx. \end{array}$$ (19)

By changing the variable $t=\frac{x}{a_{c}-\left(1-a_{c}\right)x}$ and with the help of [35], $R_{c,q}$ can be given by


$$\begin{array}{c} R_{c,q}=\frac{1}{\ln2}\int_{0}^{\infty }\left[\frac{1}{t+B_{1}}-\frac{1}{t+B_{2}}\right]\left[1-F_{A_{q}}\left(t\right)\right]dt, \end{array}$$ (20)

where $B_{1}={\left[a_{c}+\left(1-a_{c}\right)\right]}^{-1}$ and $B_{2}={\left(1-a_{c}\right)}^{-1}$. Plugging (13) into (20), $R_{c,q}$ is rewritten as


$$\begin{array}{cc} R_{c,q}= & \frac{1}{\ln2}\sum_{j_{1}=0}^{m_{q}-1}\cdots \sum_{j_{K}=0}^{m_{q}-1}\sum_{p=0}^{\Delta_{q}-1}\frac{\Lambda_{q}\left(K\right)\Gamma \left(\Delta_{q}\right)}{p!\psi_{q}^{\Delta_{q}-p}\delta_{q}^{p}}\times \int_{0}^{\infty }\left[\frac{1}{t+B_{1}}-\frac{1}{t+B_{2}}\right]e^{-\frac{\psi_{q}t}{\delta_{q}}}t^{p}dt\\ = & \frac{1}{\ln2}\sum_{j_{1}=0}^{m_{q}-1}\cdots \sum_{j_{K}=0}^{m_{q}-1}\sum_{p=0}^{\Delta_{q}-1}\frac{\Lambda_{q}\left(K\right)\Gamma \left(\Delta_{q}\right)}{p!\psi_{q}^{\Delta_{q}-p}\delta_{q}^{p}}\times \left[\int_{0}^{\infty }\frac{t^{p}e^{-\frac{\psi_{q}t}{\delta_{q}}}}{t+B_{1}}dt-\frac{t^{p}e^{-\frac{\psi_{q}t}{\delta_{q}}}}{t+B_{2}}dt\right]. \end{array}$$ (21)

To solve the integrals in (21), we use the following transformation using the Meijer G-function [36]


$$\begin{array}{c} e^{-\frac{\psi_{q}t}{\delta_{q}}}=G_{0,1}^{1,0}\left(\frac{\psi_{q}t}{\delta_{q}}|\begin{array}{c} -\\ 0 \end{array}\right). \end{array}$$ (22)

By substituting (22) into (21), $R_{c,q}$ can be expressed as


$$\begin{array}{cc} & R_{c,q}=\frac{1}{\ln2}\sum_{j_{1}=0}^{m_{q}-1}\cdots \sum_{j_{K}=0}^{m_{q}-1}\sum_{p=0}^{\Delta_{q}-1}\frac{\Lambda_{q}\left(K\right)\Gamma \left(\Delta_{q}\right)}{p!\psi_{q}^{\Delta_{q}-p}\delta_{q}^{p}}\\ & \times \left[\int_{0}^{\infty }\frac{x^{p}G_{0,1}^{1,0}\left(\frac{\psi_{q}t}{\delta_{q}}|\begin{array}{c} -\\ 0 \end{array}\right)}{t+B_{1}}dt-\frac{x^{p}G_{0,1}^{1,0}\left(\frac{\psi_{q}t}{\delta_{q}}|\begin{array}{c} -\\ 0 \end{array}\right)}{t+B_{2}}dt\right], \end{array}$$ (23)

Building upon [31, Eq. (7.811.5)] the ergodic rate of the common stream can be accurately derived as


$$\begin{array}{cc} & R_{c,q}=\frac{1}{\ln2}\sum_{j_{1}=0}^{m_{q}-1}\cdots \sum_{j_{K}=0}^{m_{q}-1}\sum_{p=0}^{\Delta_{q}-1}\frac{\Lambda_{q}\left(K\right)\Gamma \left(\Delta_{q}\right)}{p!\psi_{q}^{\Delta_{q}-p}\delta_{q}^{p}}\\ & \times \left[B_{1}^{p}G_{1,2}^{2,1}\left(\frac{\psi_{q}B_{1}}{\delta_{q}}|\begin{array}{c} -p\\ -p,0 \end{array}\right)-B_{2}^{p}G_{1,2}^{2,1}\left(\frac{\psi_{q}B_{2}}{\delta_{q}}|\begin{array}{c} -p\\ -p,0 \end{array}\right)\right]. \end{array}$$ (24)

Similarly, by using (6), the ergodic rate of the private message can be expressed as $R_{p,q}=𝔼\left[\log_{2}\left({\bar{\gamma }}_{p,q}+1\right)\right]$. Solving $R_{p,q}$ using steps similar to (23) yields


$$\begin{array}{cc} & R_{p,q}=\frac{1}{\ln2}\sum_{j_{1}=0}^{m_{q}-1}\cdots \sum_{j_{K}=0}^{m_{q}-1}\sum_{p=0}^{\Delta_{q}-1}\frac{\Lambda_{q}\left(K\right)\Gamma \left(\Delta_{q}\right)}{p!\psi_{q}^{\Delta_{q}-p}\delta_{q}^{p}}\\ & \times \left[B_{3}^{p}G_{1,2}^{2,1}\left(\frac{\psi_{q}B_{3}}{\delta_{q}}|\begin{array}{c} -p\\ -p,0 \end{array}\right)-B_{4}^{p}G_{1,2}^{2,1}\left(\frac{\psi_{q}B_{4}}{\delta_{q}}|\begin{array}{c} -p\\ -p,0 \end{array}\right)\right], \end{array}$$ (25)

where $B_{3}={\left[a_{q}+\left(1-a_{c}-a_{q}\right)\right]}^{-1}$ and $B_{4}=a_{q}^{-1}$. Using (25) and (24) into (17), we get (18). This completes the *proof of Proposition 1*.

High SNR Region: When $\varrho_{S}\to \infty$, the law of large numbers may be applied to estimate ${\tilde{\gamma }}_{c,q}^{*,\infty }\to \frac{a_{c}}{1-a_{c}}$ and ${\tilde{\gamma }}_{p,q}^{*,\infty }\to \frac{a_{q}}{1-a_{c}-a_{q}}$. The respective ergodic rate boundaries of $x_{c}$ and $x_{q}$ at $U_{q}$ can be derived as


$$\begin{array}{ccc} & & R_{c,q}^{\infty }≃\log_{2}\left(1+a_{c}/1-a_{c}\right), \end{array}$$ (26a)

$$\begin{array}{ccc} & & R_{p,q}^{\infty }≃\log_{2}\left(1+a_{q}/1-a_{c}-a_{q}\right)\text{.} \end{array}$$ (26b)

By substituting (26b) and (26a) into (17), the ergodic sum rate under high SNR circumstances may be represented as


$$\begin{array}{cc} R_{sum}^{\infty }≃ & R_{c,q}^{\infty }+\sum_{q=1}^{Q}R_{p,q}^{\infty }=\log_{2}\left(\frac{1}{1-a_{c}}\right)+\sum_{q=1}^{Q}\log_{2}\left(\frac{1-a_{c}}{1-a_{c}-a_{q}}\right). \end{array}$$ (27)

*Remark 2:* The high-SNR approximation reveals that the ergodic sum rate grows logarithmically with transmit power, while the choice of $a_{c}$ critically affects the balance between common and private message rates. This emphasizes the need for optimal power allocation in RSMA-based satellite-terrestrial networks.

## 4 SER analysis

The SER for a given modulation scheme can be expressed as [37, Eq. (37)]


$$\begin{array}{cc} S_{a,q}= & r𝔼\left\{Q\left(\sqrt{v{\bar{\gamma }}_{a,q}}\right)\right\}=\frac{r\sqrt{v}}{2\sqrt{2\pi }}\int_{0}^{\infty }\frac{e^{-\frac{v}{2}x}}{\sqrt{x}}F_{{\bar{\gamma }}_{a,q}}\left(x\right)dx, \end{array}$$ (28)

where a∈{c,p} represents either the common message (*c*) or the private message (*p*), $Q(x)=\frac{1}{2\pi }\int_{x}^{\infty }e^{-t^{2}\cdot 2}dt$ is the Gaussian Q-function, *r* and *v* are modulation-specific constants (e.g., for BPSK, *r* = 1, *v* = 2; for QPSK, *r* = 2, *v* = 1) in [38] and ${\bar{\gamma }}_{a,q}$ is the SINR for decoding either the common or private message at user *q*.

To analyze the SER, the derived SINR expressions are substituted into above equation and apply statistical methods to evaluate the expected value over the fading distribution.

### 4.1 SER of the common message

*Proposition 2:* Since all users need to successfully decode the common message, its SER is determined by the worst-performing user in the network. Using probability theory and integral approximations, The closed-form approximate expression of SER for the common message is approximated as


$$\begin{array}{cc} S_{c,q}\approx & \frac{r\sqrt{v}}{2\sqrt{2\pi }}[\sqrt{\frac{2\pi }{v}}\Phi \left(\sqrt{\frac{va_{c}}{2\left(1-a_{c}\right)}}\right)-\frac{\pi a_{c}}{2W\left(1-a_{c}\right)}\\ & \times \sum_{w=1}^{W}\frac{\sqrt{1-\xi_{w}^{2}}e^{-\frac{vS\left(\xi_{w}\right)}{2}-\frac{\psi_{q}G\left(\xi_{w}\right)}{\delta_{q}}}G{\left(\xi_{w}\right)}^{p}}{\sqrt{S\left(\xi_{w}\right)}}], \end{array}$$ (29)

where *W* is a trade-off parameter controlling complexity and accuracy, Φ(.) is the Error function, $G\left(t\right)=\frac{t+1}{\left(1-a_{c}\right)\left(1-t\right)}$, $S\left(t\right)=\frac{a_{c}\left(t+1\right)}{2\left(1-a_{c}\right)}$ and $\xi_{w}=\cos\left(\frac{2w-1}{2W}\pi \right)$.

*Proof:* Submitting (5) into (28), we have $S_{c,q}$ is given by


$$\begin{array}{cc} S_{c,q}= & \frac{r\sqrt{v}}{2\sqrt{2\pi }}\int_{0}^{\infty }\frac{e^{-\frac{v}{2}x}}{\sqrt{x}}F_{{\bar{\gamma }}_{c,q}}\left(x\right)dx=\frac{r\sqrt{v}}{2\sqrt{2\pi }}\int_{0}^{\frac{a_{c}}{1-a_{c}}}\frac{e^{-\frac{v}{2}x}}{\sqrt{x}}\left[1-F_{A_{q}}\left(\frac{x}{a_{c}-\left(1-a_{c}\right)x}\right)\right]dx. \end{array}$$ (30)

From (13), let’s rewrite the result in (30) as


$$\begin{array}{cc} S_{c,q}= & \frac{r\sqrt{v}}{2\sqrt{2\pi }}\int_{0}^{\frac{a_{c}}{1-a_{c}}}\frac{e^{-\frac{v}{2}x}}{\sqrt{x}}[1-\sum_{j_{1}=0}^{m_{q}-1}\cdots \sum_{j_{K}=0}^{m_{q}-1}\sum_{p=0}^{\Delta_{q}-1}\frac{\Lambda_{q}\left(K\right)\Gamma \left(\Delta_{q}\right)}{p!\psi_{q}^{\Delta_{q}-p}\delta_{q}^{p}}\\ & \times e^{-\frac{\psi_{q}\left(\frac{x}{a_{c}-\left(1-a_{c}\right)x}\right)}{\delta_{q}}}{\left(\frac{x}{a_{c}-\left(1-a_{c}\right)x}\right)}^{p}]dx\\ = & \frac{r\sqrt{v}}{2\sqrt{2\pi }}[\underset{I_{1}}{\underbrace{\int_{0}^{\frac{a_{c}}{1-a_{c}}}\frac{e^{-\frac{v}{2}x}}{\sqrt{x}}dx}}-\sum_{j_{1}=0}^{m_{q}-1}\cdots \sum_{j_{K}=0}^{m_{q}-1}\sum_{p=0}^{\Delta_{q}-1}\frac{\Lambda_{q}\left(K\right)\Gamma \left(\Delta_{q}\right)}{p!\psi_{q}^{\Delta_{q}-p}\delta_{q}^{p}}\\ & \times \underset{I_{2}}{\underbrace{\int_{0}^{\frac{a_{c}}{1-a_{c}}}\frac{e^{-\frac{v}{2}x}}{\sqrt{x}}e^{-\frac{\psi_{q}\left(\frac{x}{a_{c}-\left(1-a_{c}\right)x}\right)}{\delta_{q}}}{\left(\frac{x}{a_{c}-\left(1-a_{c}\right)x}\right)}^{p}dx}}]. \end{array}$$ (31)

For the first integration of (31), using [31, Eq. (3.361.1)], $I_{1}$ is given by


$$\begin{array}{c} I_{1}=\sqrt{\frac{2\pi }{v}}\Phi \left(\sqrt{\frac{va_{c}}{2\left(1-a_{c}\right)}}\right), \end{array}$$ (32)

where Φ(.) is the Error function. For the second integral, setting $t=\frac{2\left(1-a_{c}\right)x}{a_{c}}-1$ results in $x=\frac{a_{c}\left(t+1\right)}{2\left(1-a_{c}\right)}$. Hence, $I_{2}$ is given as


$$\begin{array}{c} I_{2}=\frac{a_{c}}{2\left(1-a_{c}\right)}\int_{-1}^{1}\frac{e^{-\frac{vS\left(t\right)}{2}-\frac{\psi_{q}G\left(t\right)}{\delta_{q}}}}{\sqrt{S\left(t\right)}}G{\left(t\right)}^{p}dt, \end{array}$$ (33)

where $G\left(t\right)=\frac{t+1}{\left(1-a_{c}\right)\left(1-t\right)}$ and $S\left(t\right)=\frac{a_{c}\left(t+1\right)}{2\left(1-a_{c}\right)}$.

Using the Gaussian-Chebyshev quadrature method in [39,40], we can obtain $I_{2}$ as


$$\begin{array}{c} I_{2}\approx \frac{\pi a_{c}}{2W\left(1-a_{c}\right)}\sum_{w=1}^{W}\frac{\sqrt{1-\xi_{w}^{2}}e^{-\frac{vS\left(\xi_{w}\right)}{2}-\frac{\psi_{q}G\left(\xi_{w}\right)}{\delta_{q}}}}{\sqrt{S\left(\xi_{w}\right)}}G{\left(\xi_{w}\right)}^{p}, \end{array}$$ (34)

where $\xi_{w}=\cos\left(\frac{2w-1}{2W}\pi \right)$.

Plugging (34) and (32) into (31), we will acquire (29). The *proof* is completed.

### 4.2 SER of private messages

For private messages, the SER depends on the ability of each user to successfully decode its intended signal while treating interference from other users as noise. In a like manner, employing (6), the SER for private messages can be represented as $S_{p,q}=r𝔼\left\{Q\left(\sqrt{v{\bar{\gamma }}_{p,q}}\right)\right\}$. By following a process similar to that in (29) to deduce $S_{p,q}$, we arrive at


$$\begin{array}{cc} & S_{p,q}\approx \frac{r\sqrt{v}}{2\sqrt{2\pi }}[\sqrt{\frac{2\pi }{v}}\Phi \left(\sqrt{\frac{va_{q}}{2\left(1-a_{c}-a_{q}\right)}}\right)-\frac{\pi a_{q}}{2W}\\ & \times \frac{1}{\left(1-a_{c}-a_{q}\right)}\sum_{w=1}^{W}\frac{\sqrt{1-\xi_{w}^{2}}e^{-\frac{v\tilde{S}\left(\xi_{w}\right)}{2}-\frac{\psi_{q}\tilde{G}\left(\xi_{w}\right)}{\delta_{q}}}\tilde{G}{\left(\xi_{w}\right)}^{p}}{\sqrt{\tilde{S}\left(\xi_{w}\right)}}], \end{array}$$ (35)

where $\tilde{G}\left(t\right)=\frac{t+1}{\left(1-a_{c}-a_{q}\right)\left(1-t\right)}$ and $\tilde{S}\left(t\right)=\frac{a_{q}\left(t+1\right)}{2\left(1-a_{c}-a_{q}\right)}$.

### 4.3 High SNR region

At high transmit power levels, the SER expressions simplify significantly. In the high-SNR regime ($\varrho_{S}\to \infty$), the SER can be approximated as


$$\begin{array}{cc} & S_{c,q}=rQ\left(\sqrt{\frac{va_{c}}{1-a_{c}}}\right), \end{array}$$ (36a)

$$\begin{array}{cc} & S_{p,q}=rQ\left(\sqrt{\frac{va_{q}}{1-a_{c}-a_{q}}}\right). \end{array}$$ (36b)

*Remark 3:* This shows that increasing $a_{c}$ improves the reliability of the common message, but excessive allocation can degrade private message performance.

## 5 Results and discussions

In this section, numerical simulations are presented to validate the derived formulas. The parameters for Shadowed-Rician fading can be found in Table 2, while the details for the numerical outcomes are consolidated in Table 3. We consider a baseline scenario involving two users (*Q* = 2). Furthermore, the equivalent noise power at $U_{q}$ was determined as $\sigma_{q}^{2}=N_{0}+10\log\left(BW\right)+NF$ [dBm] as referenced and the respective power allocation coefficients are $a_{1}=0.6\left(1-a_{c}\right)$ and $a_{2}=0.4\left(1-a_{c}\right)$.

**Table 2:** Table of Satellite Channel Parameters [41–43].

Shadowing	$b_{q}$	$m_{q}$	$\Omega_{q}$
Average shadowing (AS)	0.251	5	0.279
Heavy shadowing (HS)	0.063	1	0.0007

**Table 3:** Main system parameters.

Monte Carlo simulations	10^6^ iterations
Satellite type	GEO
Antennas of satellite	*K* = 2
Number of users	*Q* = 2
Power allocation of common data	$a_{c}=0.4$
Carrier frequency	$f_{c}=2$ [GHz]
Carrier bandwidth	*W* = 15 [Mhz]
Receiver noise temperature	$T=300^{\circ }K$
Speed of light	$c=3\times 10^{8}$ [m/s]
Distance from satellite to $U_{q}$	$d_{SU_{q}}=35786$ [Km]
Antenna gain at satellite	$\vartheta_{S}=53.45$ [dB]
Antenna gain at satellite and $U_{q}$	$\vartheta_{U_{q}}=4.8$ [dB]
Angular separation	$\theta_{U_{q}}=0.8^{\circ }$
3dB beamwidth	$\theta_{U_{q}3dB}=0.3^{\circ }$
Bandwidth	BW=10 [MHz]
Noise figure	NF=10 [dBm]
Thermal noise power density	$N_{0}=-174$ [dBm/Hz]

**Fig 2:**
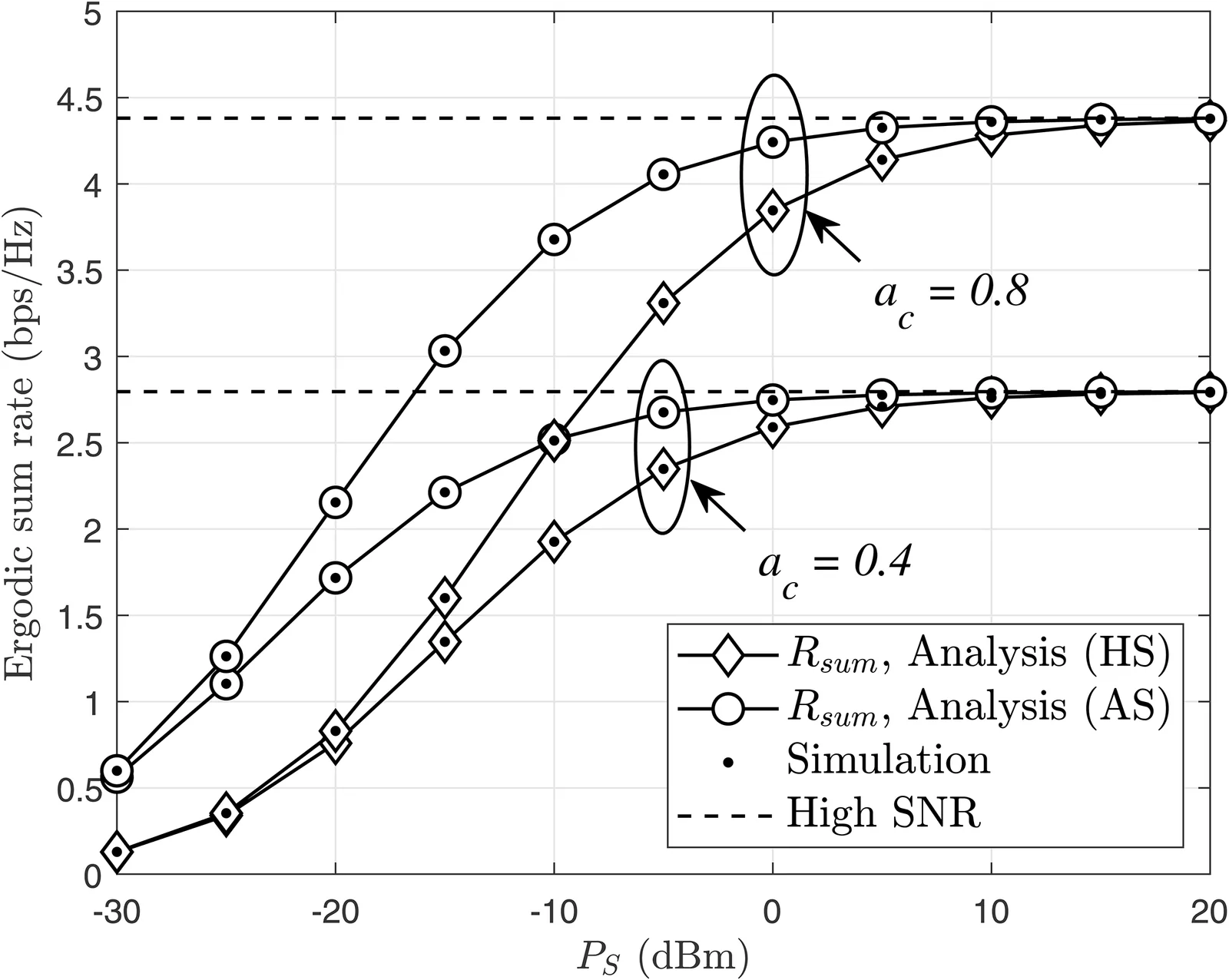
Ergodic sum rate versus $P_{S}$ for power allocation factors $a_{c}$, with *Q* = 2, *K* = 2 and $\left\{a_{1},a_{2}\right\}=\left\{\left\{0.36,0.24\right\},\left\{0.12,0.08\right\}\right\}$.

Fig 2 presents the ergodic sum rate as a function of transmit power $P_{S}$ for different values of the power coefficient $a_{c}$ under both AS and HS conditions. The results show that the simulation ergodic sum rate increases with $P_{S}$, aligning with the analytical expression in (18). The high SNR region in (27) closely matches the exact analysis at high power levels, validating its accuracy. Under AS conditions, the system achieves a significantly higher sum rate than in HS, demonstrating the impact of severe fading on performance. The choice of $a_{c}$ also plays a crucial role, as a higher $a_{c}$ initially improves the sum rate but may lead to diminishing gains at higher $P_{S}$. This suggests that optimizing $a_{c}$ is essential for maximizing system performance in satellite-terrestrial networks.

**Fig 3:**
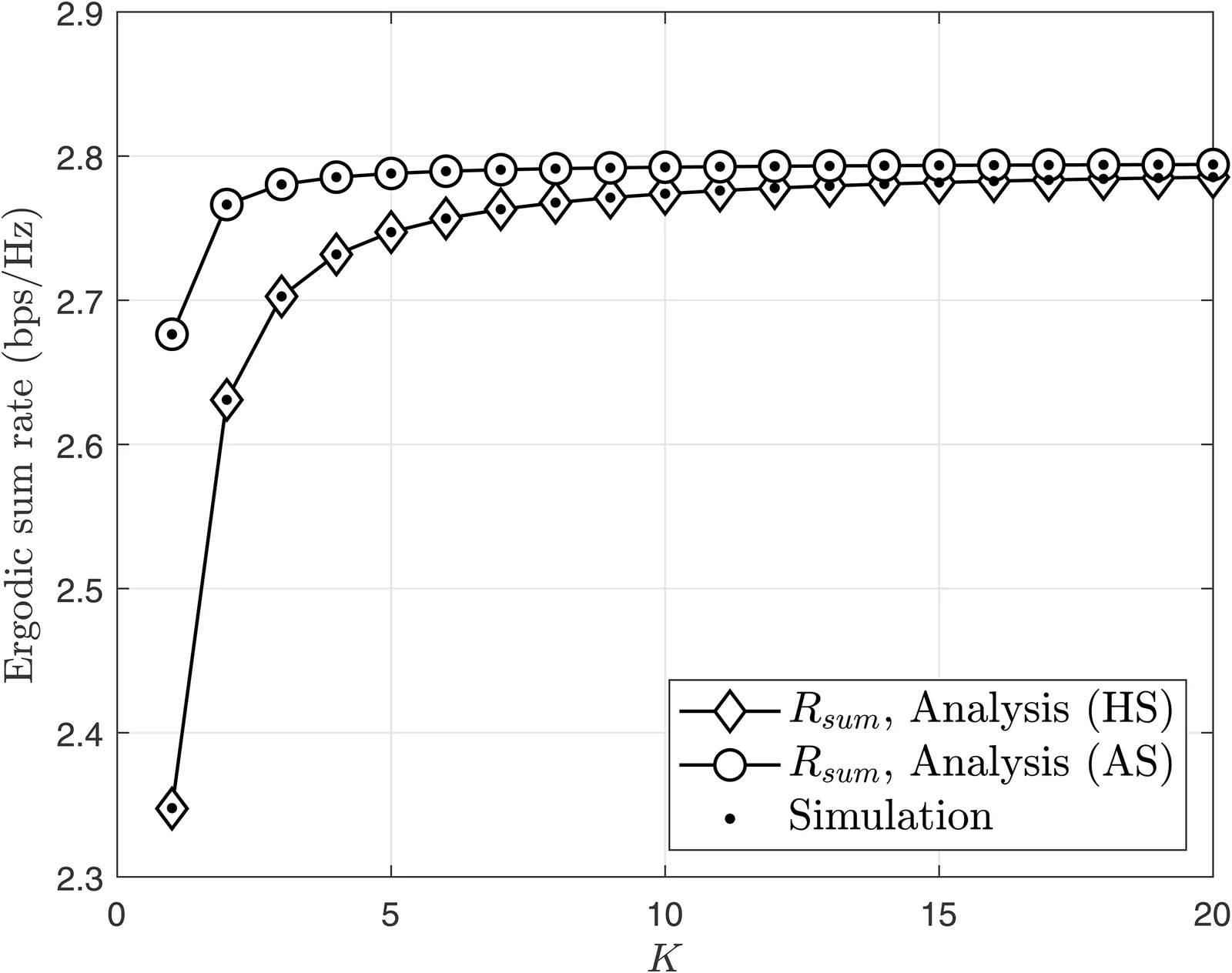
The ergodic sum rate versus *K* with increasing number of antennas at satellite, for *Q* = 2, $a_{c}=0.4$, $\left\{a_{1},a_{2}\right\}=\left\{0.36,0.24\right\}$ and $P_{S}=-5$ dBm.

Fig 3 clearly demonstrates that the ergodic sum rate increases substantially with the number of satellite antennas (*K*). The results confirm that increasing *K* provides improvements of sum rate, driven by superior beamforming gains and more effective interference mitigation. Moreover, it is evident that ergodic sum rates under AS conditions consistently outperform those under HS, owing to reduced fading effects. These findings underscore the decisive influence of channel conditions on system performance and make a compelling case for deploying additional antennas in RSMA-based satellite-terrestrial communication systems.

**Fig 4:**
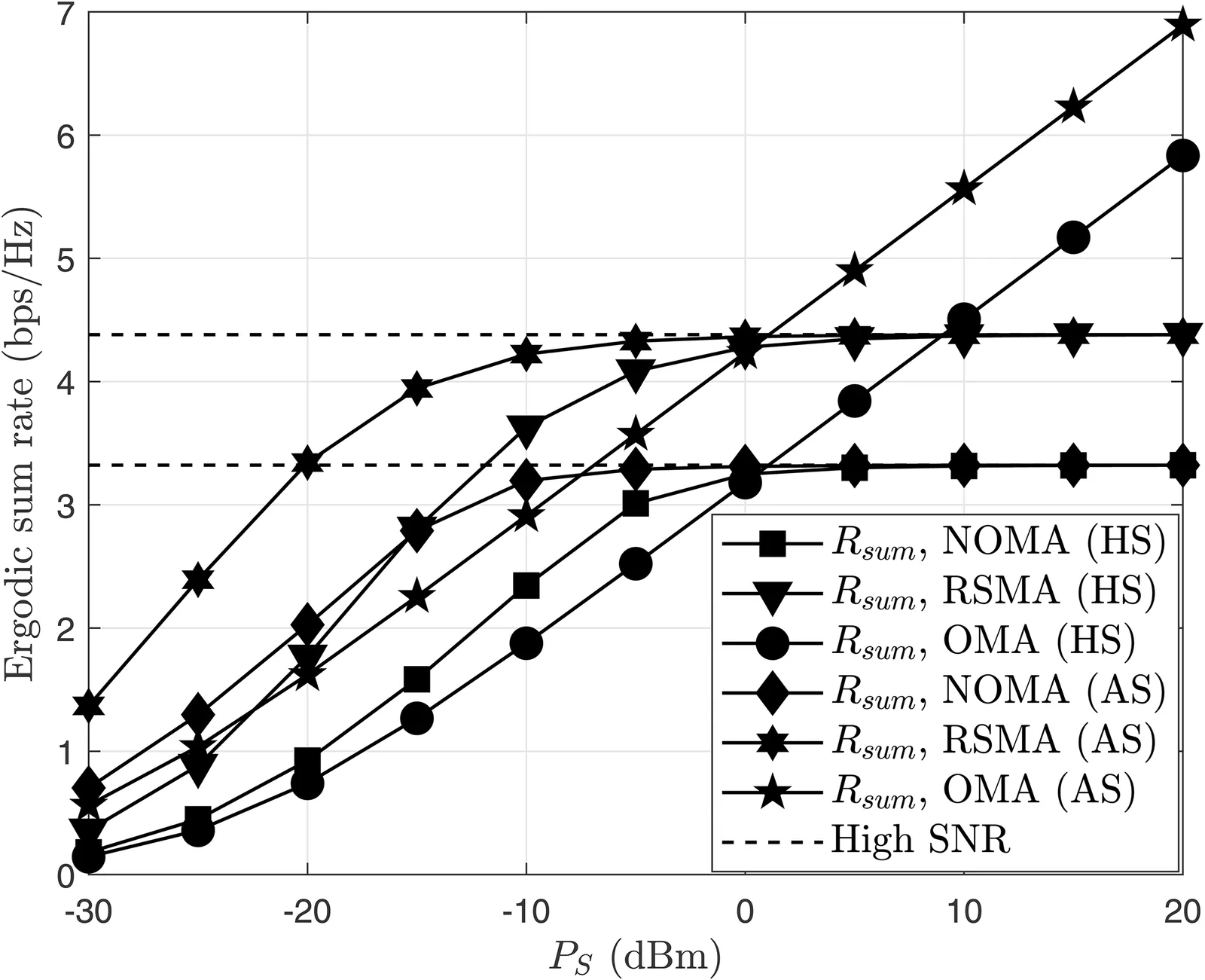
Comparison between RSMA and NOMA for the ergodic sum rate versus $P_{S}$, with *K* = 3 and $a_{c}=0.8$, *a*_1_ = 0.12, *a*_2_ = 0.08 and *Q* = 2.

Fig 4 compares the ergodic sum rate of RSMA, NOMA and OMA as the transmit power $P_{S}$ increases under AS and HS scenarios. The results show that RSMA consistently achieves a higher sum rate than NOMA at all power levels. This is because RSMA effectively manages interference by splitting messages into common and private parts, allowing for more flexible decoding at the receiver. The performance gap is more noticeable at lower $P_{S}$, where interference plays a bigger role. As $P_{S}$ increases, all schemes improve, with OMA being lower at low $P_{S}$ (from −30–0 dBm) but becoming the highest from 0 dBm on-wards due to the absence of interference saturation. It is also worth noting that OMA outperforms RSMA in the high-transmit-power region in Fig 4. This behaviour can be explained by the different interference characteristics of the considered schemes. At low and moderate transmit powers, the system performance is strongly affected by noise, and RSMA benefits from its ability to partially decode and manage interference through the common stream. Hence, RSMA achieves a higher ergodic sum rate than NOMA and OMA in this operating region. However, as the transmit power increases, the noise term becomes less significant, and the performance of RSMA becomes increasingly limited by the residual inter-user interference among the private streams. This leads to an interference-saturation effect in the high-SNR regime. By contrast, OMA allocates orthogonal time/frequency resources to different users, thereby avoiding intra-resource inter-user interference. Although this orthogonalization causes a resource-partitioning loss, the absence of inter-user interference enables OMA to achieve a higher sum rate than RSMA at sufficiently high transmit powers under the considered power allocation and channel settings. This observation does not contradict the advantages of RSMA; rather, it highlights that the relative performance of RSMA, NOMA, and OMA depends on the operating SNR regime, interference level, and resource-allocation strategy.

**Fig 5:**
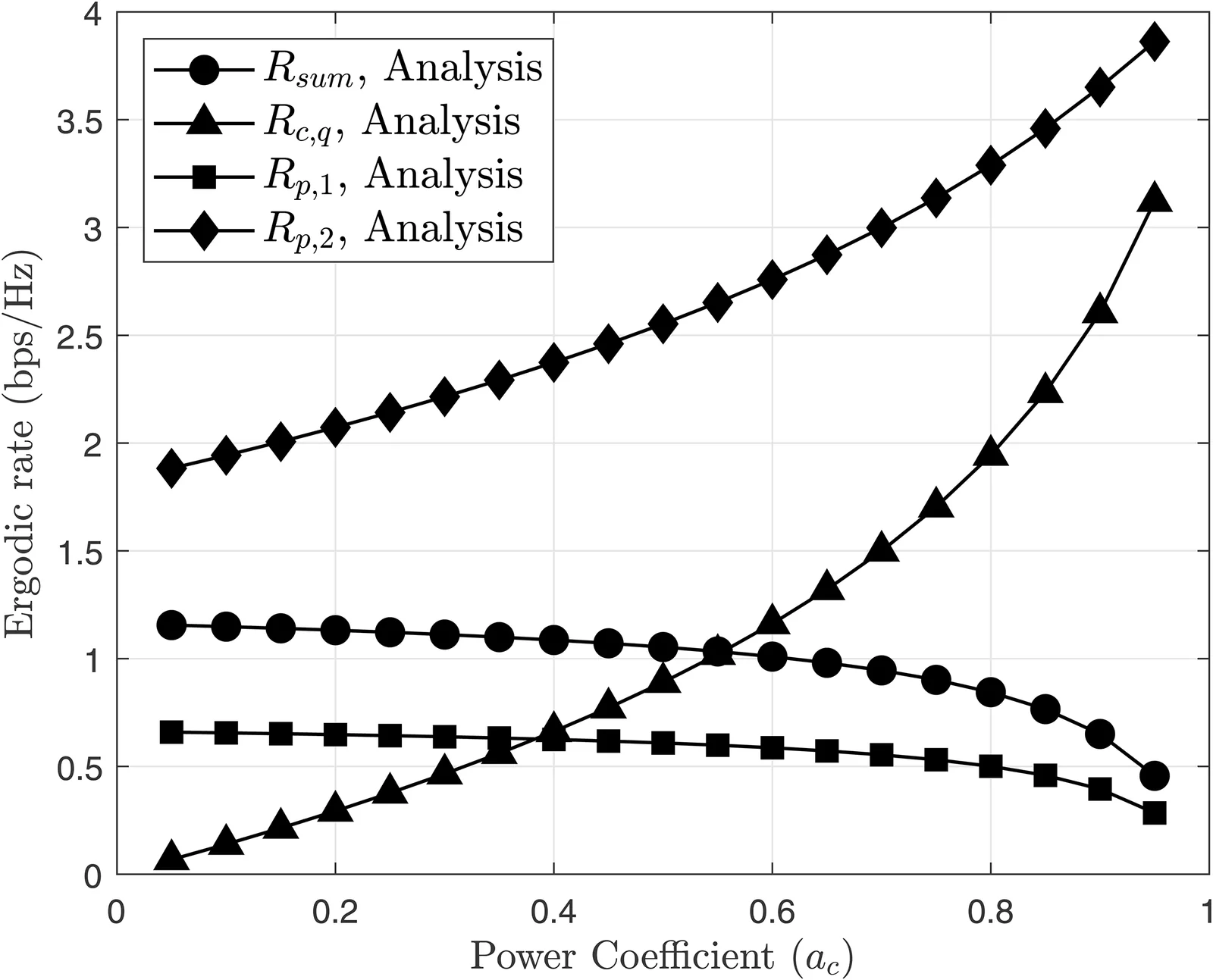
The ergodic rate versus power coefficient $\left(a_{c}\right)$, with *Q* = 2, *K* = 2, $m_{q}=1$, $b_{q}=0.063$, $\Omega_{q}=0.0007$ and $P_{S}=-10$ dBm.

Fig 5 illustrates the variation of the ergodic sum rate changes with the power coefficient $a_{c}$ for the common stream. The results indicate that increasing $a_{c}$ initially improves the sum rate, but beyond a certain point, the performance starts to decline. This happens because allocating too much power to the common stream reduces the power available for private streams, which limits overall system efficiency. The optimal value of $a_{c}$ depends on system conditions, such as the number of users and channel fading. The results also show that the sum rate is higher under AS than HS, as stronger fading in HS reduces signal quality. These findings highlight the importance of properly adjusting $a_{c}$ to balance common and private stream power for the best system performance.

**Fig 6:**
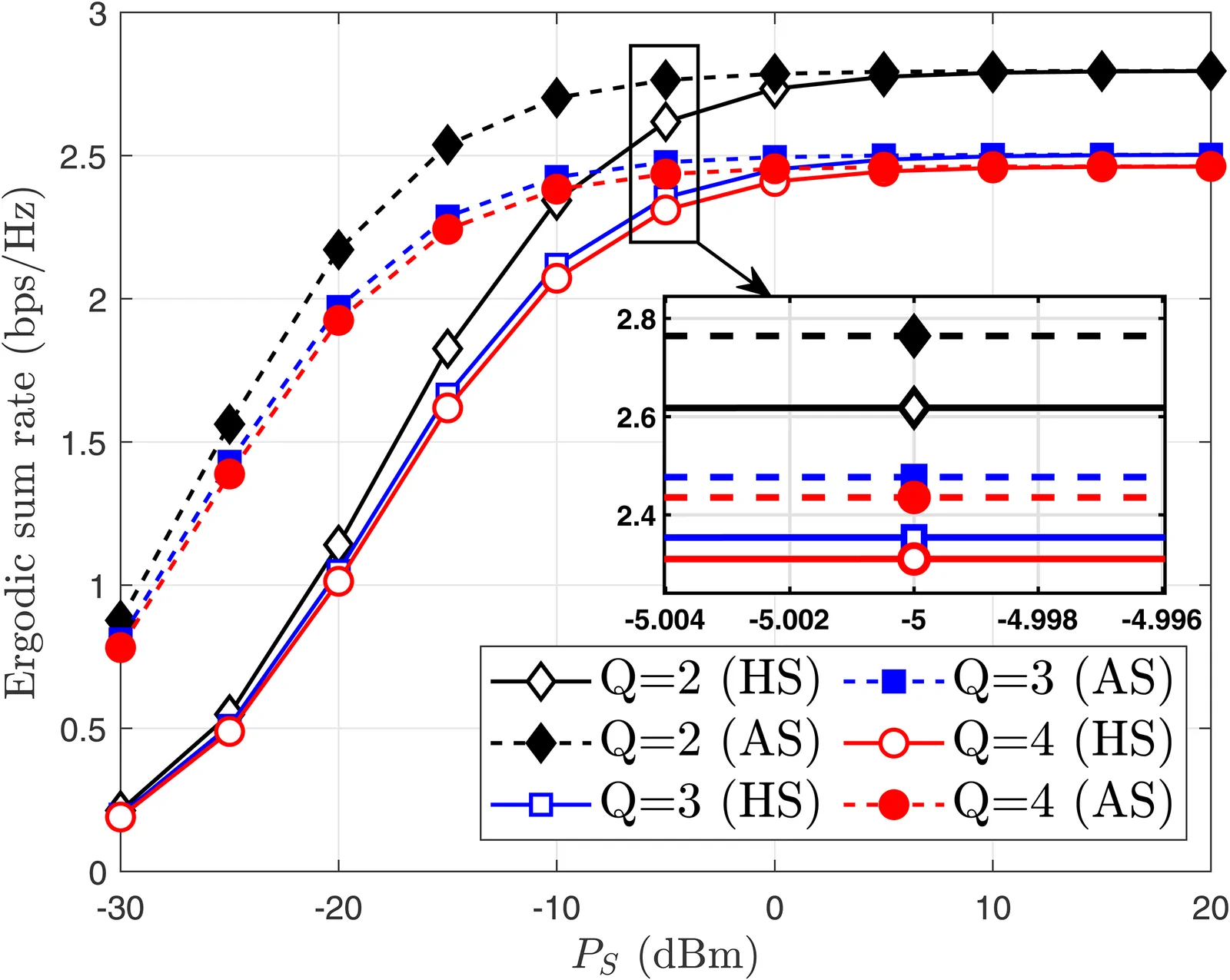
Ergodic sum rate versus $P_{S}$ different numbers of users Q∈{2,3,4} with *K* = 2 and $a_{c}=0.4$ under HS and AS environments.

Fig 6 illustrates the ergodic sum rate of the proposed RSMA scheme versus $P_{S}$ for *Q* = 2, 3, and 4 users under both HS and AS conditions. It can be observed that the ergodic sum rate decreases slightly as the number of users *Q* increases. This trend is theoretically justified since the system operates in an overloaded regime. In such scenarios, the spatial degrees of freedom are limited, which prevents the MRT beamforming from completely eliminating inter-user interference among the private streams. Furthermore, because the common stream’s rate is strictly bounded by the user experiencing the worst channel conditions, adding more users increases the probability of encountering a deeper fade, thereby compressing the common rate floor. Nevertheless, the system avoids severe outage and maintains stable connectivity across all users, confirming the scalability of the RSMA transmission framework under massive access setups.

**Fig 7:**
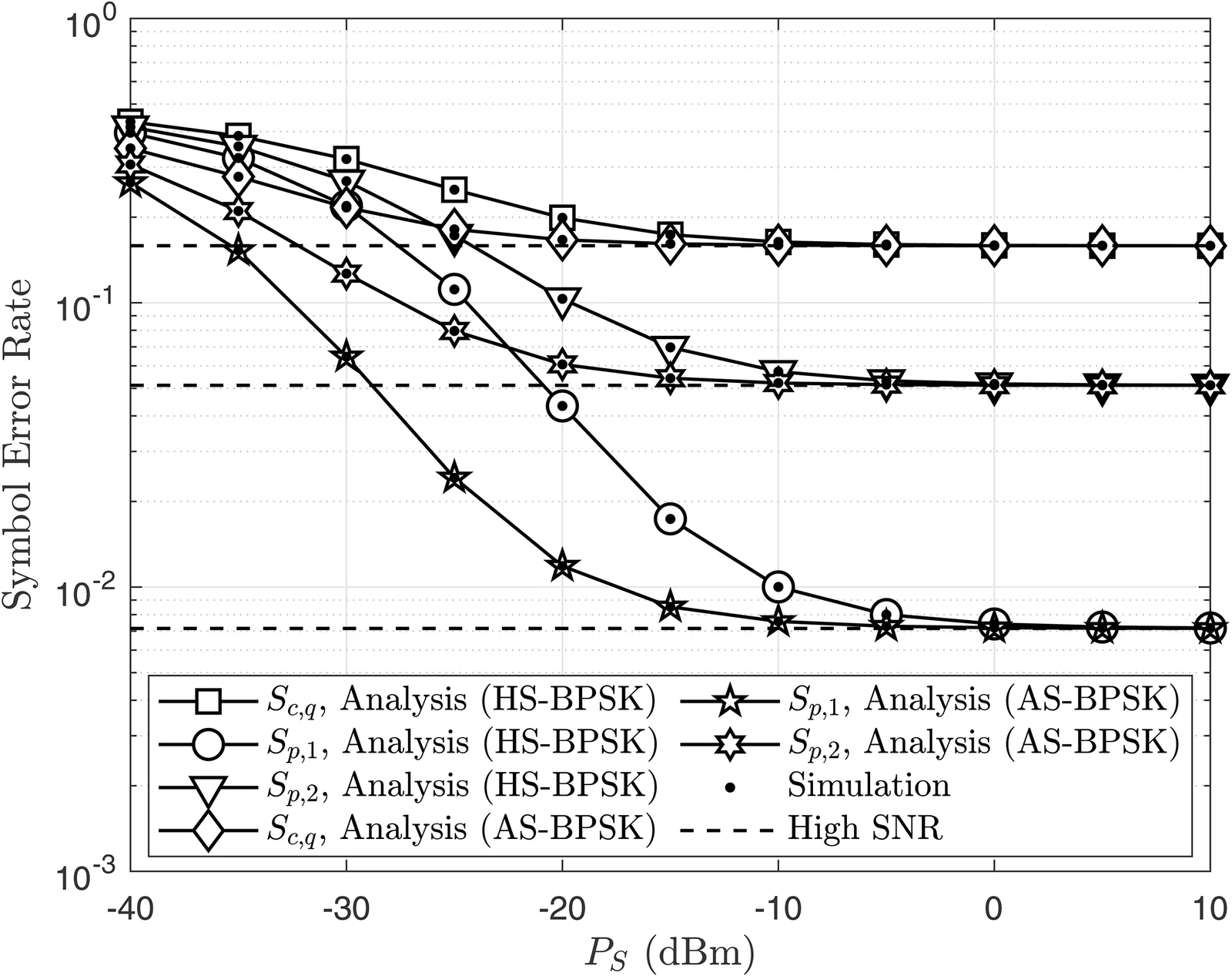
The SER versus $P_{S}$, with *Q* = 2, *K* = 4, $\left\{a_{1},a_{2}\right\}=\left\{0.48,0.32\right\}$ and $a_{c}=0.2$.

Fig 7 presents the SER versus the transmit power $P_{S}$ under different fading conditions. The results show that as $P_{S}$ increases, the SER decreases for all cases, which is expected because higher transmit power improves signal strength and reduces detection errors. However, the rate of improvement varies depending on the shadowing conditions. The SER is consistently lower under AS compared to HS since stronger fading in HS degrades the signal quality. The results also confirm that the analytical expression closely follows the simulation data, validating the accuracy of the derived model. These findings emphasize the importance of considering fading effects in system design, especially in low-power scenarios where SER is more sensitive to channel variations.

**Fig 8:**
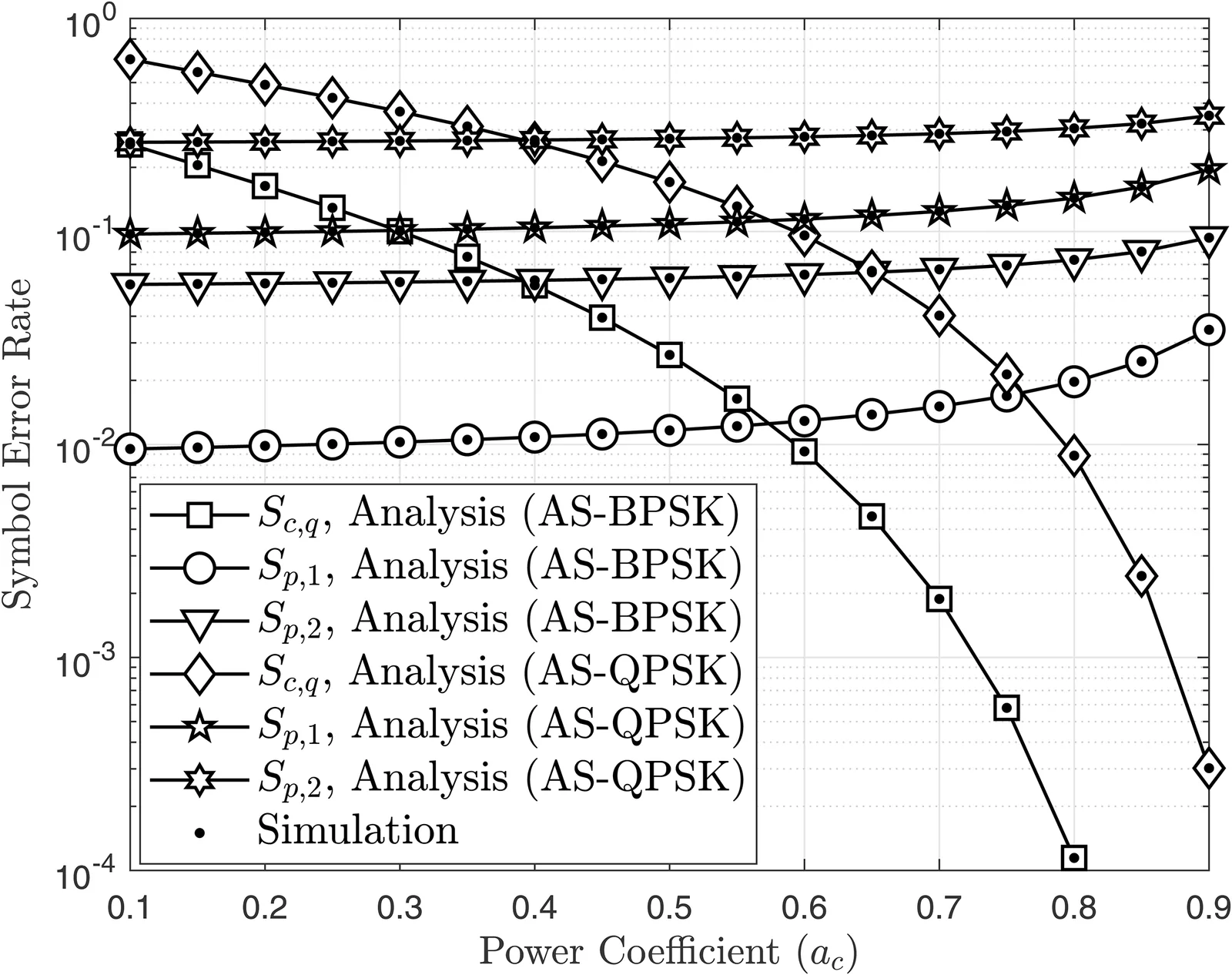
The SER versus power coefficient $\left(a_{c}\right)$, with *Q* = 2, *K* = 6, $m_{q}=5$, $b_{q}=0.251$, $\Omega_{q}=0.279$ and $P_{S}=-20$ dBm.

Finally, Fig 8 illustrates the SER versus the power coefficient $a_{c}$ for the common stream under BPSK and QPSK modulation schemes. The results show that as $a_{c}$ increases, the SER consistently decreases. This trend indicates that allocating more power to the common stream improves the overall detection accuracy, reducing symbol errors. Additionally, the SER for BPSK is lower than for QPSK at the same $a_{c}$, which aligns with the fact that BPSK is more robust to noise and fading compared to QPSK. The SER under AS is also lower than under HS, showing that severe fading conditions significantly degrade performance. These findings highlight the importance of adjusting $a_{c}$ appropriately and considering modulation type to enhance reliability in satellite-terrestrial communication systems.

## 6 Conclusion

This paper investigated the performance of an RSMA-enabled satellite-terrestrial communication system under shadowed-Rician fading. Closed-form analytical expressions were derived for the ergodic sum rate and symbol error rate, providing useful insight into the effects of power allocation, fading severity, and the number of satellite antennas on system performance. The numerical results verified the accuracy of the proposed analysis and showed that RSMA can achieve notable performance gains over conventional NOMA in terms of spectral efficiency and interference management, particularly under challenging fading conditions. Nevertheless, it is important to emphasize that the analytical framework developed in this work relies on several idealized benchmark assumptions. In particular, perfect CSI is assumed to enable tractable MRT beamforming and closed-form performance analysis. In practical satellite-terrestrial systems, CSI may be imperfect due to channel estimation errors, limited or quantized feedback, feedback delay, beam pointing mismatch, and CSI aging. Such imperfections may reduce the accuracy of beamforming and power allocation, thereby degrading the achievable ergodic sum rate and SER performance. In addition, the present analysis assumes ideal SIC at the receivers. In practical implementations, SIC may be affected by decoding errors, receiver impairments, and residual interference. Residual SIC can limit the effectiveness of common-stream cancellation and may increase the interference observed during private-stream detection, leading to performance degradation, especially in interference-limited operating regimes. Moreover, the considered analytical model does not explicitly capture dynamic satellite mobility. While the GEO-based benchmark provides a useful reference case, more dynamic satellite scenarios, such as LEO systems, may experience time-varying propagation distances, Doppler shifts, rapid beam movement, and frequent channel variations. These effects can make CSI acquisition, beam tracking, and adaptive resource allocation significantly more challenging. Therefore, the results presented in this paper should be interpreted as a theoretical benchmark for RSMA-enabled satellite-terrestrial networks under idealized operating conditions.

Future work will extend the proposed framework by incorporating imperfect CSI, residual SIC, hardware impairments, and dynamic satellite mobility. Robust RSMA transmission strategies, adaptive beam tracking, and low-complexity power allocation algorithms will also be investigated to improve the applicability of the proposed framework to practical satellite-terrestrial deployments.
